# Diabetes mellitus in sub-saharan Africa during the COVID-19 pandemic: A scoping review

**DOI:** 10.1371/journal.pone.0305112

**Published:** 2024-07-08

**Authors:** Wenceslaus Sseguya, Silver Bahendeka, Sara MacLennan, Nimesh Mody, Aravinda Meera Guntupalli

**Affiliations:** 1 Institute of Applied Health Sciences, University of Aberdeen, Aberdeen, United Kingdom; 2 Department of Internal Medicine, St Francis Hospital Nsambya, Kampala, Uganda; 3 Mother Kevin Postgraduate Medical School, Uganda Martyrs University, Kampala, Uganda; 4 Institute of Medical Sciences, University of Aberdeen, Aberdeen, United Kingdom; Universal Scientific Education and Research Network, UNITED KINGDOM

## Abstract

**Background:**

The COVID-19 pandemic impacted the healthcare and outcomes of individuals with various chronic diseases. However, there is a paucity of data on the impact of the COVID-19 pandemic on diabetes mellitus (DM) in low-resource settings. To address this, we conducted a scoping review to explore the literature published on diabetes-related COVID-19 outcomes and care during the COVID-19 pandemic in countries of sub-Saharan Africa.

**Methods:**

We applied our search strategy to PubMed, Web of Science, CINAHL, African Index Medicus, Google Scholar, Cochrane Library, Scopus, Science Direct, ERIC and Embase to obtain relevant articles published from January 2020 to March 2023. Two independent reviewers were involved in screening the retrieved articles. Data from eligible articles were extracted from quantitative, qualitative and mixed-methods studies. Quantitative evidence was summarised using descriptive statistics, while a thematic framework was used to identify and categorise themes from qualitative evidence.

**Results:**

We found 42 of the retrieved 360 articles eligible, mainly from South Africa, Ethiopia and Ghana (73.4%). The incidence of DM among COVID-19 cases was 13.7/1,000 person-days observation. COVID-19 was associated with increased odds of death (OR 1.30–9.0, 95% CI), hospitalisation (OR 3.30–3.73: 95% CI), and severity (OR: 1.30–4.05, 95% CI) in persons with DM. Challenges in caring for DM during the pandemic were inadequate patient self-management, difficulties in healthcare access, and inadequate healthcare resources.

**Conclusion:**

The COVID-19 pandemic was characterised by a high incidence of DM in persons infected with severe acute respiratory syndrome coronavirus 2 (SARS-CoV-2) and high COVID-19-associated mortality, severity, and hospitalisation among people persons with DM. The pandemic also created difficulties in DM self-management and worsened the quality of DM care services. Policymakers should devise preventive and management strategies for DM during emerging and re-emerging infectious disease epidemics and outbreaks, given that such occurrences are increasingly frequent in the region.

## Introduction

Global evidence suggests that the coronavirus disease 2019 (COVID-19) resulted in a worldwide surge in mortality, morbidity, and disability, which predominantly occurred among older adults and individuals with chronic disease conditions [1,2]. COVID-19 has been reported to worsen diabetes mellitus (DM) clinical outcomes in particular and DM care in general [3–6]. However, little is known about this context in low- and middle-income countries, particularly in sub-Saharan Africa (SSA).

While SSA is host to about 24 million of the estimated 537 million people with DM globally, the region records the highest rate of DM-related premature mortality [7]. Furthermore, SSA is predicted to experience the highest rate of rise in DM prevalence than any other region by 2040, depicting the magnitude of a growing threat [7]. Compared to other world regions, a comprehensive linkage between diabetes and COVID-19 is inadequately researched in SSA. This could mean a limited understanding of the scale of the COVID-19 impact on DM outcomes and care in a region where the health systems struggle to meet healthcare demands. To address this gap, we conducted a scoping review to collate knowledge in this area that supports evidence-based policy consideration and stimulates future research in SSA.

To understand the scope of this research area, we reviewed published quantitative, qualitative and mixed methods literature to assess the influence of the COVID-19 pandemic on DM in SSA. More specifically, we assessed the incidence of DM among COVID-19 cases, outcomes of COVID-19 and their predictors among people with pre-existing DM (PWDM), and challenges in caring for DM during the COVID-19 pandemic.

## Methods

### Study design

We report our scoping review in line with the Preferred Reporting Items for Systematic Reviews and Meta-analyses extension for Scoping Reviews (PRISMA-ScR) (S1 Table). The initial protocol for this scoping review is reposited with Open Science Framework [https://doi.org/10.17605/OSF.IO/9JCKF].

### Data sources and search strategy

We relied on regional and international databases to collate evidence from as many relevant sources as possible. We searched ten electronic databases, i.e., PubMed, Web of Science, Cumulative Index to Nursing and Allied Health Literature (CINAHL), African Index Medicus, Google Scholar, Cochrane Library, Scopus, Science Direct, Education Resource Information Centre (ERIC) and Embase. Our search strategy was guided by the SPIDER (Sample population, Phenomenon of Interest, Design, Evaluation and Research type) framework outlined by Cooke et al. [8] to identify relevant literature from qualitative and mixed methods studies. Additionally, to capture relevant literature from quantitative studies, we enriched our search strategy by incorporating appropriate elements of the PICO (Population, Intervention, Comparison and Outcome) framework [9]. The detailed search strategy (S2 Table) used various search strings that were appropriately applied across the citation databases. An initial search across all databases was conducted in May 2022 and later updated using the same search strategy in March 2023 to include any relevant records published during most of the pandemic period that the WHO declared a “Public Health Emergency of International Concern” [10]. This also opened up possibilities for including any emerging evidence on different ‘waves’ of COVID-19 infection and interventions. All retrieved records were merged into a single MS^®^ Excel file for subsequent removal of duplicates and screening.

### Selection criteria

The retrieved records were screened for eligibility through two stages: an initial review of title and abstract, and a subsequent full-text review of articles to be considered for final inclusion. We included peer-reviewed full-text articles published in English between 01 January 2020 and 22 March 2023 reporting on COVID-19 and DM in SSA. The classification of SSA considered was that adopted by the World Bank [12]. We excluded studies for which full-texts were not available (after failed attempts to get them from authors), duplicate articles, review articles, and articles published as multicountry studies involving countries outside SSA without disaggregation of country-specific data. WS and AMG independently conducted an initial screening for the title and abstract, which SB reviewed, resolving any disagreements in screening decisions. The same approach was applied for full-text screening. We defined agreement as a matching decision independently held by the reviewers involved in the screening process.

### Data extraction and management

Data variables of interest from the selected articles were extracted and charted in the extraction form. The data extraction form was developed by WS and reviewed by AMG, SB, and SM. It was then tested by WS and AMG with two randomly selected articles from each set of quantitative, qualitative, and mixed methods studies for appropriateness. Further revisions were made and continuously updated throughout the data extraction process by WS and AMG. Data extraction and charting were conducted by WS and independently reviewed by AMG and SB. The extracted data variables were DM incidence among COVID-19 cases, DM prevalence, COVID-19 outcomes among COVID-19 PWDM, predictors of COVID-19 outcomes in PWDM, predictors of new-onset DM in COVID-19 cases, patient challenges in caring for DM, health worker challenges in caring for PWDM, health facility challenges in providing DM services, and changes in the organisation of DM services (S4 Table).

### Data synthesis

We grouped the included studies by study design, i.e., quantitative, qualitative and mixed-methods studies. Under each group, we summarised the study settings, phenomena, population, outcomes measured, challenges in DM care reported and related broad findings. We used tables to present the summaries of COVID-19 outcomes, their predictors and DM incidence, as well as categorise the various reported DM care challenges during the pandemic.

## Results

### Selection and characteristics of included studies

**Fig 1:**
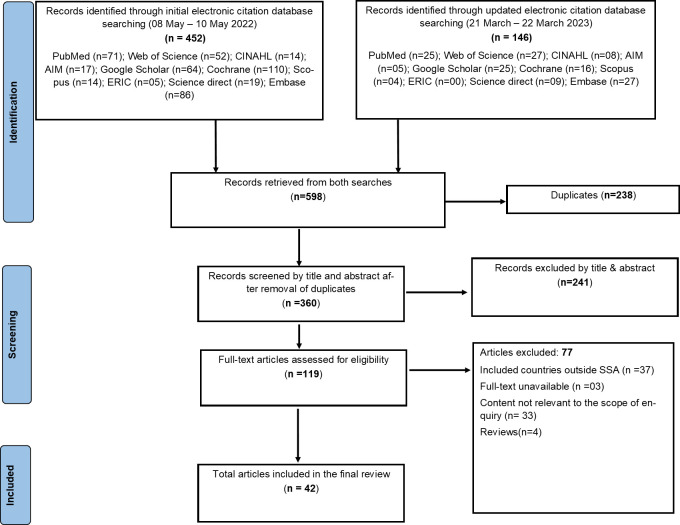
PRISMA-ScR diagram illustrating the selection process of the 42 included articles.

A total of 360 unique records were retrieved from database searches, 42 [11–51] of which were eligible for final inclusion (Fig 1). Inter-reviewer reliability analysis using Cohen’s kappa showed substantial agreement between reviewers at title and abstract screening (k = 0.626, p<0.01) and moderate agreement at full-text screening (k = 0.545, *p<0*.*01*).

Quantitative evidence was mainly represented by epidemiological observational studies (n = 38), which included cross-sectional (n = 26), cohort (n = 11) and case-control (n = 01) studies that were primarily from South Africa (n = 14). The majority of these observational studies were retrospective (n = 28), published between 2021 and 2022 (n = 34), with sample sizes ranging from 30 [17] to 3,460,932 [16]. Two studies from South Africa [13] and Ghana [32] represented qualitative evidence. Two studies, all from South Africa, represented mixed-methods evidence, with a sample size of 18 [22] and 544 [42]. Evidence of DM incidence among COVID-19 cases and COVID-19 outcomes and their predictors among PWDM were exclusively reported from quantitative studies (Table 1). Conversely, evidence of challenges in caring for DM during the COVID-19 pandemic came mainly from qualitative and mixed-methods studies (Table 2). The prevalence proportion of pre-existing DM among COVID-19 cases [11,12,19,20,23,24,30,33–38,40,44,46–50,52] ranged from 2.0% [24] to 51% [38].

**Table 1:** Studies reporting DM in COVID-19 cases, COVID-19 outcomes and their predictors.

Refereence (Year)	Country	Study design	Sample	DM in COVID-19 cases			COVID-19 outcomes				COVID-19 outcome predictors in DM		
				DM prevalence	DM incidence	COVID-19 mortality		COVID-19 severity	COVID-19 hospitalisation	mortality		hospitalisation	new-onset DM
[11] (2021)	South Africa	Cross-sectional	1,376 COVID-19-admitted patients	**X**		**X**							
[12] (2021)	South Africa	Cross-sectional	9,305 persons with DM diagnosed with COVID-19	**X**	**X**	**X**			**X**	**X**		**X**	
[23] (2021)	South Africa	Cohort	261 patients visiting emergency centre hospitalisation with an initial negative PCR test	**X**		**X**							
[34] (2022)	South Africa	Cross-sectional	10,149 health workers	**X**				**X**					
[45] (2022)	South Africa	Cross-sectional	568 admitted patients with laboratory-confirmed SARS-CoV2 infection			**X**							
[47] (2021)	Ethiopia	Cohort	2,617 COVID-19 admitted patients with positive RT-PCR	**X**		**X**		**X**					
[48] (2021)	South Africa	Cross-sectional	242 hospitalised adults (> = 18years) with laboratory-confirmed COVID-19	**X**									
[49] (2021)	Cameroon	Cohort	313 patients admitted with suspicion or confirmed COVID-19	**X**				**X**					
[50] (2022)	Nigeria	Cross-sectional	200 admitted COVID-19 patients	**X**									
[51] (2021)	Ethiopia	Case-control	147 patients admitted with confirmed COVID-19 RT-PCR diagnosis			**X**							
[15] (2020)	Democratic Republic of Congo	Cohort	141 hospitalised patients with RT-PCR confirmed COVID-19						**X**				
[16] (2021)	South Africa	Cohort	3,460,932 patients with PCR-confirmed COVID-19			**X**				**X**			
[17] (2021)	Congo	Cross-sectional	30 SARS-CoV 2-infected patients with pre-existing DM			**X**							
[18] (2022)	South Africa	Cohort	236 hospitalised patients >13years with laboratory-confirmed SARS-CoV 2 infection			**X**				**X**			
[19] (2022)	Ethiopia	Cross-sectional	686 patients admitted with RT-PCR confirmed diagnosis of COVID-19	**X**				**X**					
[52] (2021)	Ethiopia	Cohort	1,345 patients admitted with RT-PCR confirmed of COVID-19	**X**									
[20] (2020)	Ghana	Cross-sectional	50 hospital-admitted COVID-19 diagnosed patients	**X**		**X**							
[21] (2021)	South Africa	Cross-sectional	1,447 patients admitted with confirmed COVID-19 and pre-existing or newly diagnosed DM			**X**				**X**			
[24] (2022)	Ghana	Cross-sectional	2,334 PCR confirmed COVID-19 patients	**X**					**X**				
[27] (2022)	Uganda	Cohort	664 hospitalised patients with confirmed COVID-19			**X**							
[28] (2021)	Gabon	Cross-sectional	837 COVID-19 hospitalised patients					**X**					
[30] (2021)	South Africa	Cross-sectional	100 patients who died of COVID-19	**X**									
[31] (2022)	Côte d’Ivoire	Cross-sectional	67 COVID-19 infected persons			**X**							
[33] (2022)	South Africa	Cross-sectional	1,861 SARS CoV 2 admitted patients	**X**									
[35] (2022)	Kenya	Cohort	1,792 admitted COVID-19 patients	**X**					**X**				
[36] (2023)	Ghana	Cross-sectional	175 adult patients hospitalised with COVID-19	**X**									
[37] (2022)	Ethiopia	Cross-sectional	463 SARS CoV2 positive patients aged ≥18	**X**		**X**		**X**					
[38] (2022)	South Africa	Cross-sectional	413 ICU admitted COVID-19 patients aged ≥18	**X**		**X**							
[39] (2022)	South Africa	Cross-sectional	188,292 private health insurance patients who tested positive for COVID-19						**X**				
[40] (2022)	Senegal	Cross-sectional	67,608 community population	**X**		**X**							
[41] (2022)	Ethiopia	Cohort	304 severe COVID-19 hospitalised patients		**X**								**X**
[43] (2022)	Ethiopia	Cross-sectional	244 COVID-19-admitted patients with DM diagnosis		**X**								**X**
[44] (2022)	South Africa	Cross-sectional	3,217 COVID-19 hospitalised patients	**X**	**X**								
[46] (2022)	Ethiopia	Cohort	552 COVID-19 hospitalised patients	**X**		**X**							

**Table 2:** Studies reporting challenges of caring for DM during the COVID-19 pandemic.

Reference (Year)	Country	Study design	Sample	Categories of themes reported			
				DM patient challenges	Health worker challenges	Health facility challenges	DM care service reorganisation
[13] (2020)	South Africa	Qualitative report	Community Health Worker teams			**X**	**X**
[14] (2022)	Ethiopia	Cross-sectional	576 adult type 2 DM patients	**X**			
[22] (2022)	South Africa	Mixed methods	28 primary care health workers and patients living with NCDs (type 2 DM and hypertension)	**X**	**X**	**X**	**X**
[25] (2021)	Ghana	Cross-sectional	157 DM patients aged 20 years and over	**X**			
[26] (2021)	Rwanda	Cross-sectional	52 young adults with type 1 DM	**X**			
[29] (2020)	Nigeria	Cross-sectional	374 persons aged 15 years and older	**X**			
[32] (2023)	Ghana	Qualitative	18 healthcare professionals and health facility administrators	**X**	**X**	**X**	**X**
[42] (2022)	South Africa	Mixed methods	544 type 2 DM patients in routine care	**X**			**X**

### Incidence of DM and predictors in COVID-19 cases

The DM incidence rate of 13.7/1,000 person-days observation was reported [41], with a reported median duration from admission to DM occurrence of 11 (IQR 7,13) days [41]. The proportion of new-onset DM among COVID-19 cases [12,41,43,44] ranged from 7.3% [44] to 31.1% (95% CI: 25.4, 37.4) [43], with 88% of cases manifesting as type 2 DM [43]. Predictors of new-onset DM in COVID-19 cases were age over 41 years (adjusted Hazard Ration, HR = 2.54, 95% CI: 1.15, 5.57) [41], being male (adjusted Odds Ratio, OR = 2.9, 95% CI:1.2,7.1) [43] and urban residence (adjusted HR = 2.49, 95% CI: 1.12, 5.52) [41]. The evidence did not specify predictors for different types of DM or indicate specific incidence statistics for different waves of COVID-19 infection.

### Outcomes of COVID-19 and their predictors in PWDM

#### COVID-19 mortality and predictors

The proportion of COVID-19 deaths in PWDM [17,18,20,21,23,31,37,38,45,51] ranged from 5.3% [21] to 66% [38], with new-onset DM reported to account for 19% of these deaths [45]. The odds of PWDM dying of COVID-19 [11,12,16,27,40,45–47,51] ranged from 1.31 (adjusted Relative Risk 95% CI: 0.77, 2.23) [40] to 9.0 (adjusted OR 95% CI:1.73, 47.07) [27]. Predictors of COVID-19 mortality in PWDM [12,16,21] were age per 5-year ageing interval (OR 1.33, 95%CI: 1.30, 1.37) [12], glycated haemoglobin (HbA1c) of 7.0% to 8.9% (HR 1.81, 95% CI:1.39, 2.35), HbA1c greater than 8.9% (HR 1.60, 95% CI: 1.27, 2.0) [16], being male (OR 1.70, 95% CI: 1.5, 1.92 to OR 2.05, 95% CI: 1.07, 3.93)[12,21], using insulin treatment (OR 1.49, OR 95% CI: 1.27, 1.74 to OR 2.25, OR 95% CI: 1.05, 4.85)[12,21], and admission hyperglycaemia (random capillary blood glucose  > 10 mmol/l; OR:4.24, 95% CI:1.12, 16.0) [18]. No specific COVID-19 mortality figures were reported for the different types of DM or variations in mortality across the various waves of COVID-19 infection.

#### COVID-19 severity and predictors

The proportion of COVID-19 severity recorded in PWDM [19,28,37,47] ranged from 16.1% [28] to 33.1% [37]. The odds associated with COVID-19 severity in PWDM ranged from 1.30 (adjusted OR 95% CI: 1.2, 1.5) [34] to 4.05 (OR 95% CI: 1.12, 14.15) [49]. The evidence did not report specific COVID-19 severity data by type of diabetes, rural or urban, and by different waves of COVID-19 infection.

#### COVID-19 hospitalisation and predictors

Evidence on COVID-19 hospitalisation [12,15,24,35,39] showed that 17% of hospitalised COVID-19 cases had pre-existing DM [15], and reported odds of COVID-19 hospitalisation in PWDM of 3.6 (OR 95% CI:3.27, 3.94) [39]. Pre-existing DM was associated with general hospital admission (OR 3.73, 95% CI: 3.53, 3.94) [12], intensive care unit admission (adjusted OR 3.30, 95% CI: 1.94, 560) [35], and longer duration of hospitalisation (B = 1.37, 95% CI = 0.99–1.88) [24]. The reported duration of COVID-19 hospitalisation in PWDM was 15 days, with odds of achieving clinical recovery of 0.549 (aOR 95% CI:0.337,0.894) [52]. Predictors of COVID-19 hospitalisation in PWDM were age per 5-year ageing interval (OR 1.15, 95% CI: 1.13, 1.17), being male (OR 1.41, 95% CI: 1.29, 1.54), and insulin treatment (OR:1.39, 95% CI:1.24, 1.57) [12]. No data on types of diabetes, rural settings or individual COVID-19 waves of infection was reported regarding COVID-19 hospitalisation in PWDM.

### Challenges of caring for DM during the COVID-19 pandemic

Eight studies (Table 2), with qualitative (n = 02) [13,32], mixed-methods (n = 02) [22,42] and quantitative evidence (n = 04) [14,25,26,29], reported challenges of caring for DM during the COVID-19 pandemic. The evidence was categorised into DM patient challenges, health worker challenges, health facility challenges, and reorganisation of DM care services (S3 Table).

#### Patient challenges in caring for DM

Fear and worry among patients were reported during the COVID-19 pandemic which were attributed to information on adverse consequences of COVID-19 circulated through media. Studies show that 82.8% of PWDM aged over 20 were ‘more careful about taking medication than usual’, 33.8% worried “about people with diabetes being characterised as a risk group”, 42% expressed worry “they would be overly affected if infected with coronavirus due to diabetes”, and 49.7% worried about ‘not being able to manage diabetes if infected with coronavirus’ [25]. Reduced food access, intake and meal frequency were also reported due to the pandemic’s imposed lockdowns and restrictions [25,26]. Forty-two percent of PWDM aged 20 years and over reported to have eaten less than usual [25]. Among persons with type 1 diabetes, 57.7% and 65.4% reported a reduction in their meal frequency and limited access to food respectively [26]. The change in food intake and meal frequency affected daily dose adjustments and medication scheduling, which was mentioned in those on insulin treatment [25,26]. A drop in family income due to COVID-19-related disruptions in trade and work was also reported in 80.8% of people with type 1 DM [26]. Additionally, the pandemic lockdowns and restrictions on social distancing were reported to affect the lifestyle of people with DM [14,26]. Adherence to physical activity recommendations was reported in only 26.4% of people with type 2 DM during the pandemic, although relatively higher proportions were in rural than urban settings (adjusted OR 1.95, 95% CI: 1.63, 3.27, p<0.05) [14]. In people with type 1 DM, 43.1% reported reduced physical activity during the pandemic lockdown [26]. Challenges in accessing health centres and services in PWDM were also reported [22,26,29,32]. There was a reported increase (81.8%) in travel by foot to DM centres during the lockdown among persons with type 1 DM, with some reporting to have “faced problems with law enforcement when trying to access their DM supplies and attend DM healthcare appointments, in ways they had not prior experienced” [26]. Type 1 DM patients also reported an increased occurrence of episodes of hypoglycaemia during the COVID-19 pandemic that worried them [26]. For the general DM population, the cancellation of routine non-communicable disease clinics [22] and the closure of some health facilities [29] caused disruptions in access to services, with more difficulty reported in accessing essential medicines due to scarcity and high costs [29]. DM patients also reported to face increased waiting time and extended clinic review appointments [22] due to healthcare changes implemented in the wake of the COVID-19 pandemic.

#### Health worker challenges in providing DM care

Health workers reported a general increase in workload and overcrowding of clinics caused by the high patient demand for health services during the pandemic, which reduced quality care time with patients [22]. There was also fear and secondary stigmatisation of health workers associated with their COVID-19 infection risk, which affected their interactions with DM patients [22]. Health workers also reported low morale and declining motivation at their workplaces due to unsupportive management and management’s unwillingness to make innovative changes to address pandemic working conditions [22,32]. The pandemic was also reported to exacerbate problems of poor patient information management due to COVID-19 precautions that made it difficult for health workers to perform standard clinical assessments on patients for fear of cross-infection [13,32].

#### Challenges faced by health facilities in delivering DM services

Health facilities faced challenges ranging from shortages of DM medicines and supplies due to COVID-19-related disruptions in the supply chain and rationing [32]; to reduced availability of healthcare workers at workstations due to prioritisation of COVID-19 activities and quarantining of COVID-19-infected and exposed health workers [32]; and the limited infrastructure to accommodate the high patient numbers within social distancing measures and COVID-19 prevention guidelines [32]. These contributed to the cancellation of routine NCD clinics, the closure of some health facilities, and a general decrease in DM patient turn-up [22,32]. Studies also reported that health facilities recorded a higher proportion of PWDM with poorly controlled DM [11,21]. Evidence shows that 73.2% of DM patients had HbA1c greater than 8.0%, the majority (78.6%) of whom were from rural hospitals [11]. A similar study also reported a median HbA1c of 10% (IQR 8, 12), with 86.5% having had HbA1c greater than 7.0% [21]

#### Reorganisation of DM care services

In response to COVID-19 disruptions and challenges, there was reorganisation in health services, including DM patient care delivery and clinic management. This included delivery of medicines to the homes of the patients to reduce staff workload as well as the risk of infection, adopting a clinic booking system to decongest health facilities, and utilisation of Telehealth to maintain communication between health workers and patients [13,22,26]. Two studies reported the use of community health workers in delivering medicines to patients and monitoring risk factors in the community to have reduced the workload of health centre staff [13,22]. These changes were also perceived by patients as time-saving and having reduced their risk of exposure to coronavirus infection [13]. However, some patients reported that the use of a clinic booking system caused lengthy clinic review appointment schedules and that home delivery was associated with stigma within their community [13]. Nonetheless, the involvement of community health workers was reported to improve relationships of community health workers with linkage health facilities [13]. A study among type 1 DM reported relying on Telehealth using telephones, WhatsApp and short message services (SMS) to communicate between patients and health workers in seeking medical opinions, receiving updates on supplies and troubleshooting during emergencies [26].

## Discussion

This review found 42 studies relating to DM during the COVID-19 pandemic published from 13 of 48 SSA countries between 2020 and 2023. The majority were quantitative (38/42), retrospective (28/42) and cross-sectional studies (26/42). Studies in South Africa, Ethiopia and Ghana contributed more than a third of the records included in the review (33/42). The populations studied were patients with COVID-19, patients with DM, patients with comorbid DM and COVID-19, health workers, community health workers, and health facility administrators. To the best of our knowledge, this is the first scoping review in SSA to investigate COVID-19 outcomes with DM and the challenges of DM care during the COVID-19 pandemic.

Evidence from 34 quantitative studies presented data on DM incidence among COVID-19 cases, as well as COVID-19 mortality, severity, and hospitalisation, and their predictors in PWDM. We observed a high incidence of DM among COVID-19 cases in SSA during the COVID-19 pandemic, occurring in up to 31% of COVID-19 cases with an incidence rate of 13.7/1,000 person-days. It has been observed that infection with COVID-19 may trigger the development of new-onset DM, especially in certain demographics. Specifically, COVID-19-infected individuals who reside in urban areas, are males and are over 41 years of age have a high likelihood of over twofolds of developing onset DM, typically manifesting as type 2 DM.

Conversely, we observe an association of DM with COVID-19 mortality, severity, and hospitalisation. PWDM were found to have higher odds of being hospitalised with severe COVID-19 symptoms and succumbing to COVID-19. In this regard, our review indicates that PWDM were up to nine times more likely to succumb to COVID-19, up to four times more likely to experience a severe case and up to three times more likely to require hospitalisation. Furthermore, older age, being male, poor glycaemic control, and use of insulin were all independently associated with an increased likelihood of COVID-19 death and hospitalisation in PWDM. The predictors of COVID-19 severity were not reported.

A set of eight qualitative, quantitative and mixed-methods studies provided evidence of the challenges of caring for DM during the COVID-19 pandemic drawn from the perspective of DM patients, health workers and health facilities. These studies provided data on various COVID-19 pandemic challenges that were experienced, how they impacted DM care, and the changes made in response. The studies reveal that the COVID-19 pandemic manifested in various ways that included the institution of COVID-19 lockdowns and travel restrictions, the fear created by information on COVID-19’s consequences, the increased number of COVID-19 cases demanding healthcare, the need to adhere to COVID-19 precautions and policy guidelines, and the prioritisation of COVID-19 over other diseases. These created several challenges, which, from the patient’s perspective, mainly related to poor self-management, limited accessibility of health services, and limited affordability of health services and basic needs. From the health worker’s perspective, the challenges related to increased workload, COVID-19 stigmatisation, and increased risk of COVID-19 cross-infection. From the health facility perspective, challenges related to the increased shortage of healthcare resources, poor health information management, and a high proportion of poorly controlled PWDM.

The 42 studies reveal the complexity of life for a COVID-19-infected person and a DM patient during the COVID-19 pandemic in SSA, which was characterised by considerable vulnerabilities and challenges that threatened their survival. Even when the evidence did not allow for an adequate understanding of variations of these complexities in DM types, settings and across different waves of COVID-19 infection, we notice the significant threat that COVID-19 poses in worsening the DM incidence and care burden of PWDM in SSA. This deadly impact is a major concern in the region and reveals the extent to which COVID-19 has established itself as an important epidemiological driver of the DM burden in SSA. Our scoping review highlights the vulnerabilities of different population segments to poor disease outcomes and draws the need to understand and prioritise intervention responses that are informed by consideration of inequalities in the distribution of disease risk factors and outcomes during pandemics and widespread epidemics.

Looking at the DM care challenges presented in our studies, we can interpret the COVID-19 pandemic to have been a period associated with an unprecedented burden on DM patients and healthcare resources. Unlike reports in regions elsewhere [53,54], the measures instituted by various SSA countries in response to the threat of COVID-19 resulted in numerous disruptions at various levels of society and across different sectors. From our review, we observe that these disruptions manifested in limiting travel, loss of income, increased costs of living, and increased healthcare demands amidst reduced healthcare resources, all of which contributed to the deterioration of DM self-management and quality of DM care rendered. While these limitations were encountered by PWDM in general, people with type 1 DM may have experienced deeper suffering owing to their distinct vulnerabilities [55].

Our study noted a poor overall reporting of rural-urban variations, and the limited evidence suggests that rural settings grappled with poor DM management, impacting rural dwellers with DM disproportionately. The case studies illustrating the successful reorganisation of DM care service delivery during the pandemic could provide insights for planning ahead of future pandemics. For instance, healthcare managers and policymakers could develop protocols for identifying and managing special groups and ensuring access to essential DM care services during widespread emergencies. They could also scale up telemedicine and virtual pharmacies throughout their nations to reduce infection risk for vulnerable patients and to ensure continuity of DM care management. DM patients could strengthen their voices through patient groups to increase advocacy, active involvement, and representation in intervention planning and implementation processes at various levels.

## Limitations

While this review provides reliable information by scoping various research types and sources, some limitations exist. Our review highlights an inequitable representation of DM research in SSA countries, with 35 of 48 SSA countries not represented and more than a third of the studies coming from three countries, which limits the generalisability of findings. Secondly, we only included peer-reviewed literature, which may have excluded some valuable literature sources such as manuscripts and institutional archives published by institutions or organisations. Nonetheless, we were able to use more sensitive and broader terms across carious databases that captured broad aspects of COVID-19 outcomes and DM care challenges during the pandemic reported through different methodological designs.

## Conclusions

This scoping review identified high incident diabetes in persons infected with SARS-CoV-2, and high COVID-19-associated mortality, severity, and hospitalisation. Furthermore, the COVID-19 pandemic created difficulties in diabetes self-management and access to essential services among PWDM and worsened shortages in DM care resources. Overall, SSA struggled to manage DM effectively during the pandemic, and our study underscores the need for consideration of DM among its healthcare priorities. Policymakers should devise preventive and management strategies for DM during emerging and re-emerging infectious disease epidemics, given that such epidemics and outbreaks are more frequent in the region.

## Supporting information

S1 Table Preferred Reporting Items for Systematic Reviews and Meta-Analyses extension for Scoping Reviews (PRISMA-ScR) checklist. (DOCX)

S2 Table Detailed database Search Strategy. (DOCX)

S3 Table Major themes that emerged from qualitative, quantitative, and mixed methods studies. (DOCX)

S4 Table Detailed data extraction of included articles. (DOCX)
