# Supplementary material for: Diabetes mellitus in sub-saharan Africa during the COVID-19 pandemic: A scoping review
Source: PLoS One. 2024 Jul 8;19(7):e0305112. doi: 10.1371/journal.pone.0305112 (PMC11230555; doi:10.1371/journal.pone.0305112)
Supplement: S3 Table — (DOCX) [file pone.0305112.s003.docx]

**S3 Table. Major themes on diabetes care challenges that emerged from qualitative, quantitative, and mixed methods studies.**

| **Major theme** | **Sub-theme** | **Evidence summary** |
| --- | --- | --- |
| Patient challenges in caring for DM | Difficulties in self-management | Several studies reported patient difficulties in self-management of diabetes:   - 82.8% of PWDM aged over 20 were ‘more careful about taking medication than usual’, 33.8% worried “about people with diabetes being characterised as a risk group”, 42% expressed worry “they would be overly affected if infected with coronavirus due to diabetes”, and 49.7% worried about ‘not being able to manage diabetes if infected with coronavirus’ (Ephraim RKD et al., 2021) - Reduced food intake and meal frequency due to limited access to food during the COVID-19 pandemic   - *42.0% of patients reported reduced meal frequency (Ephraim RKD et al., 2021)*   - *57.7% of patients reported a reduction in daily meal frequency (Habineza JC et al., 2021)* - Reduced physical activity engagement during the COVID-19 pandemic   - *43.1% of type 1 DM patients reported reduced physical activity (Habineza JC et al., 2021)*   - *Low adherence to physical activity recommendations was reported among persons with type 2 DM (Abate, Ferede & Mekonnen, 2022)* - Type 1 diabetes patients experience increased episodes of hypoglycaemia during the COVID-19 pandemic (Habineza, et al., 2021) |
|  | Limited affordability of health services and basic needs | Several studies reported affordability issues due to an increase in the cost of medication amidst reduced individual and household income during the pandemic.   - There was an increase in the cost and scarcity of medicines during the pandemic   - *high medicine costs due to shortages from disruption of supply chains (Tagoe et al., 2023)*   - *essential medicines became scarce and costly for the patients to afford (Awucha et al., 2020)* - COVID-19 trade disruptions and lockdowns reduced individual or household income   - *80.8% of young adults with diabetes reported a drop in family income (Habineza JC et al., 2021)* |
|  | Limited accessibility to healthcare | Studies reported health service access difficulties during the pandemic as increased waiting time, and decreased transport options, particularly during multiple lockdowns.   - Increased clinic waiting time and extended scheduling of clinic review appointments   - *Patients crowd outpatient departments from early morning, hoping to be seen early so they can go to their workplaces. However, this is not always possible because doctors attend to inpatients before seeing outpatients, resulting in long clinic waits (Tagoe et al., 2023).* - Difficulties in accessing healthcare facilities   - *There was a 16.2% decrease in the use of motorised transport during the COVID-19 pandemic and an 81.8% increase in foot travel among type 1 DM patients (Habineza et al., 2021)*   - *Type 1 DM reported to “faced problems with law enforcement when trying to access their DM supplies and attend DM healthcare appointments, in ways they had not prior experienced” (Habineza et al., 2021)* - Cancellation of routine diabetes care clinics and services |
| Health worker challenges | Increased workload | Studies reported a general increase in the workload of health workers during the COVID-19 pandemic   - The high additional demand for COVID-19 services during the COVID-19 pandemic amidst inadequate health worker numbers created a high workload for healthcare workers Delobelle et al., 2022) - There was a limited number of diabetes specialists to prioritise diabetes care during the pandemic rationing of human resources for health (Tagoe et al., 2023). |
|  | Increased risk and fear of COVID-19 cross-infection | Studies reported hesitancy of health workers to attend healthcare stations   - Health workers were hesitant to attend to patients due to fear of COVID-19 infection (Delobelle AP et al., 2022) - Healthcare management was unwilling and unsupportive in implementing innovative changes to address COVID-19 infection risk and safety among health workers (Tagoe et al., 2023) - Adherence to COVID-19 precautions for fear of infection made it difficult for health workers to perform standard clinical assessments on DM patients, which also led to inadequate capture of patient information (Tagoe et al., 2023) |
|  | COVID-19 Stigmatisation of health workers | There was reported secondary stigmatisation of health workers   - Health workers were perceived as COVID-19 carriers due to their constant involvement in COVID-19 care and were avoided by patient with other diseases and in communities (Delobelle et al., 2022) |
| Health facility challenges | Limited physical space for clinic activities | The studies reported the COVID-19 pandemic to have resulted in increased demand for healthcare resources and worsening shortages.   - The available health facility spaces were overwhelmed by high patient numbers and the need for social distancing, and spaces for some clinics were used up. Diabetes clinics had limited space to operate. (Tagoe et al., 2023) |
|  | Increased shortage of human resources | - Many health workers were deployed in COVID-19 management and surveillance roles, which reduced the number available to attend to other healthcare demands (Tagoe et al., 2023) - COVID-19 infection among health workers and quarantining of exposed health workers reduced the availability of the health workforce. Some health centres closed due to a lack of health workers after COVID-19 infection (Awucha et al., 2020) |
|  | Increased shortage of medicines and medical supplies | - There were worsened shortages in supplies of diabetes medicines and medical supplies due to disruptions in supply chains and rationing (Tagoe et al., 2023). |
|  | Increased proportion of uncontrolled DM | Studies reported increased proportion of uncontrolled DM during the pandemic   - 73.2% of DM patients had HbA1c greater than 8.0%, the majority (78.6%) of whom were from rural hospitals (Mash et al., 2021). - A median HbA1c of 10% (IQR 8, 12), with 86.5% having had HbA1c greater than 7.0% (Van der Westhuizen et al., 2021). |
| Reorganisation of diabetes care delivery | Home delivery of patient medicines | Studies reported home delivery of medicines to patients as an intervention in response to COVID-19 pandemic challenges of patient accessibility, risk of infection and fear of travel.   - Home delivery of medication to reduce infection risk, and combat fear of infection among people with diabetes while mitigating the health system access challenges faced by patients due to shutdown of transport facilities during multiple lockdowns.   - Home delivery of patient medical supplies was introduced (Brey Z et al., 2020; Delobelle AP et al., 2022; David JN et al., 2022) - Studies (n=03) reported that community health workers were empowered to provide community monitoring and follow-up of chronic diseases, including diabetes.   - - Engagement of community health workers (Brey Z et al., 2020; Delobelle AP et al., 2022; David JN et al., 2022) |
|  | Change in clinic management | A study reported the cancellation of open walk-in non-communicable disease clinic days and the creation of new methods of booking systems to manage the number of patients visiting the clinic at a particular time to avoid overcrowding.   - Cancellation of the routine noncommunicable disease clinics (Delobelle AP et al., 2022) - Institution of clinic booking system to manage patient appointments (Delobelle AP et al., 2022) - Use of Telehealth using telephones, WhatsApp and SMS for patients to link up with the diabetes care team at the diabetes centre (Habineza et al., 2021) |
