# Supplementary material for: Diabetes mellitus in sub-saharan Africa during the COVID-19 pandemic: A scoping review
Source: PLoS One. 2024 Jul 8;19(7):e0305112. doi: 10.1371/journal.pone.0305112 (PMC11230555; doi:10.1371/journal.pone.0305112)
Supplement: S4 Table — (DOCX) [file pone.0305112.s004.docx]

**S4 Table.** **Detailed data extraction of included articles**

| **Study ID** | **Author** | **Year** | **Title of publication** | **Journal** | **doi** | **Country of publication** | **Study design** | **Study location** | **Study period** | **Study Timeline** | **Phenomenon under study** | **Assessment methods/data collection** | **Study population** | **Sample size** | **Primary outcome** |
| --- | --- | --- | --- | --- | --- | --- | --- | --- | --- | --- | --- | --- | --- | --- | --- |
| 1 | Mash RJ, et al. | 2021 | Evaluation of patient characteristics, management and outcomes for COVID-19 at district hospitals in the Western Cape, South Africa: descriptive observational study | BMJ Open | 10.1136/bmjopen-2020-047016 | South Africa | Observational cross-sectional study | rural & urban district hospitals, South Africa | March 2020 - June 2020 | Retrospective | - Prevalence of comorbidities - Predictors of COVID-19 mortality | Semi-structured qualitative interviews (guide) | Patients admitted with COVID-19 | 1,376 | - 25.2% had diabetes (20.3% among rural) - Type 2 diabetes (AOR 1.84, 1.24 - 2.73, 95%CI) was independently associated with a higher risk of death. - 73.2% (n=272) had uncontrolled diabetes (HbA1c>8%), 78.6% from rural hospitals |
| 2 | Dave JA, et al. | 2021 | Risk factors for COVID-19 hospitalisation and death in people living with diabetes: A virtual cohort study from Western Cape Province South Africa | Diabetes Research and Clinical Practice | 10.1016/j.diabres.2021.108925 | South Africa | Observational cross-sectional study | rural & urban district hospitals, western Cape, South Africa | March 2020 - July 2020 | Retrospective | - Prevalence of new-onset diabetes - Predictors of hospitalization and death | hospital patient records | Persons living with diabetes diagnosed with COVID-19 | 9,305 | - 11.3% of the diabetes cases were newly diagnosed during the COVID-19 episode. - Diabetes had a high risk for COVID-19 hospital admission (OR:3.73, 95%CI 3.53,3.94) and mortality (OR:3.01,95%CI: 2.76,3.28) - Insulin use was associated with increased risk for hospitalisation (OR:1.39, 95% CI:1.24,1.57) and mortality (OR:1.49, 95% CI:1.27,1.74) - Metformin was associated with reduced risk for hospitalisation (OR 0.662, 95% CI:0.55,0.71) and mortality (OR 0.77, 95% CI:0.64;0.92) - Being male increased risk of COVID-19 hospitalisation (OR 1.41, 95%CI: 1.29,1.54) and mortality (OR 1.70, 95%CI: 1.51, 1.92) - Age per 5-year interval was associated with increased risk of COVID-19 hospitalisation (OR 1.15, 95%CI: 1.13,1.17) and mortality (OR 1.33, 95%CI: 1.30,1.37) |
| 3 | Van Hoving DJ, et al. | 2021 | Demographic and clinical characteristics of hospitalised patients under investigation for COVID-19 with an initial negative SARS-CoV-2 PCR test result | African Journal of Emergency Medicine | 10.1016/j.afjem.2021.09.002 | South Africa | Observational Cohort | Rural and urban hospitals in South Africa | April 2020 - June 2020 | Retrospective | - Prevalence of comorbidities - Proportion of deaths among persons with diabetes | close-ended structure questionnaire | patients visiting emergency centre with an initial negative PCR test result and requiring hospitalisation | 261 | - Diabetes (19.2%) was among the common comorbidities in the admitted patients. - 37.5% of deaths occurred in persons with diabetes |
| 4 | Ratshikhopha E. et al. | 2022 | Disease severity and comorbidities among health worker COVID-19 admissDeions in South Africa: a retrospective analysis | International Journal of Environmental Research and Public Health | 10.3390%2Fijerph19095519 | South Africa | Observational cross-sectional study | Urban hospitals, South Africa | March 2020 - December 2021 | Retrospective | - Prevalence of comorbidities - Predictors of COVID-19 severity | Self-administered questionnaire | health workers | 10,149 | - 27.6% of COVID-19 cases had diabetes. - comorbid diabetes (aOR: 1.3, 95%CI 1.2-1.5) was associated with a higher risk for disease severity. |
| 5 | Claassen N, et al. | 2022 | Experiencing COVID-19 at a large district level hospital in Cape Town: a retrospective analysis of the first wave | Southern African Journal of Infectious Diseases | 10.4102/sajid.v37i1.317 | South Africa | Observational cross-sectional study | Urban hospital in Cape Town, South Africa | April 2020 - August 2020 | Retrospective | - characteristics of survivors and deceased COVID-19 hospitalised patients - predictors of death | Data abstraction tool | Admitted patients with laboratory-confirmed a positive SARS-CoV 2 infection test | 568 | - 51% of deaths occurred in patients with diabetes. - Diabetes was associated with a higher risk of death (OR 2.7, 95% CI: 1.8 - 3.9) - 19% of deaths in patients with diabetes were new onset |
| 6 | Abraha HE, et al. | 2021 | Clinical features and risk factors associated with morbidity and mortality among patients with COVID-19 in northen Ethiopia | International Journal of Infectious Diseases | 10.1016/j.ijid.2021.03.037 | Ethiopia | Observational cohort | rural and urban hospitals in Northern Ethiopia | May 2020 - October 2020 | Retrospective | - Characteristics of COVID-19 cases - Predictors of mortality | Patient medical records | RT-PCR positive COVID-19 admitted patients | 2,617 | - 3.1% of all cases and 18.4% of severe cases had comorbid diabetes. - Diabetes was associated with higher in-hospital mortality among COVID-19 patients (uRR: 7.73, 95% CI: 2.58-23.12 |
| 7 | Kaswa R, et al. | 2021 | Clinical outcomes of hospitalised COVID-19 patients at Mthatha Regional Hospital, Eastern Cape, South Africa: a retrospective study | South African Family Practice | 10.4102/safp.v63i1.5253 | South Africa | Observational cross-sectional study | Rural hospital, Eastern Cape South Africa | March 2020 - July 2020 | Retrospective | - Characteristics of clinical outcomes | Self-administered web-based electronic questionnaire | Hospitalised adult (>=18years) with laboratory-confirmed COVID-19 | 242 | - Diabetes occurred in 36.8% of the cases - Diabetes was the commonest comorbidity associated with higher mortality |
| 8 | Mbarga FN, et al. | 2021 | Clinical profile and factors associated with COVID-19 in Yaounde, Cameroon: a prospective cohort study | PLOS ONE | [10.1371/journal.pone.0251504](https://doi.org/10.1371/journal.pone.0251504) | Cameroon | Observational cohort | Urban hospitals in Cameroon | April 2020 - July 2020 | Prospective | - Clinical characteristics of cases - Predictors of COVID-19 severity | Excel spreadsheet used to manually collect data from clinical health records | Patients admitted with suspicion or confirmed COVID-19 | 313 | - 5.8% of cases had diabetes - Diabetes was associated with increased COVID-19 severity (OR: 4.05, 95% CI 1.12,14.15; *p=0.01*) |
| 9 | Kwaghe VG, et al. | 2022 | Clinical characteristics and outcome of the first 200 patients hospitalised with COVID-19 at a treatment centre in Abuja, Nigeria: a retrospective study | Pan African Medical Journal | 10.11604/pamj.2022.41.118.26594 | Nigeria | Observational cross-sectional study | Urban hospital in Abuja, Nigeria | March 2020 - June 2020 | Retrospective | - Characteristics of cases | Review of published health reports | COVID-19 patients admitted at an isolation centre | 200 | 18.5% of the cases had diabetes |
| 10 | Leulseged TW, et al. | 2021 | Predictors of death in severe COVID-19 patients at millennium COVID-19 care centre in Ethiopia: a case-control study | Pan African Medical Journal | 10.4269%2Fajtmh.21-1270 | Ethiopia | Observational case-control | Urban hospital in Ethiopia | June 2020 - September 2020 | Retrospective | - Predictors of clinical outcomes | Qualitative semi-structured interviews / topic guide | Patients admitted with confirmed RT-PCR diagnosis of COVID-19. case: patients admitted whose outcome was death control: admitted patients whose outcome was recovery and discharged alive | 147 | - Having diabetes was associated with higher death outcomes than those with no diabetes (53.3% vs 46.7%, *p=0.001*) - Diabetes patients exhibited higher odds of dying compared to those with no diabetes (AOR:3.26, 95% CI:1.35,7.87), *p=<0.01*. |
| 11 | Brey Z, et al. | 2020 | Home delivery of medication during Coronavirus disease 2019,Cape Town, South Africa | African Journal of Primary Health Care & Family Medicine | 10.4102%2Fphcfm.v12i1.2449 | South Africa | Qualitative | Urban community setting in Cape Town, South Africa | 2020 | Retrospective report | - Home delivery of medication for chronic disease patients | Data records of the national COVID-19 DATCOV surveillance system | Community Health Workers | unspecified | - 46.2% of the delivery target was achieved - The intervention was affected by incomplete, outdated and missing patient records and failure to reach registered phone contacts. - Perceived opportunities were improved relationships of community health workers with linkage facilities and improved risk factor tracking. - Perceived threats were stigma associated with home delivery |
| 12 | Abate HK, et al. | 2022 | Adherence to physical exercise recommendations among type 2 diabetes patients during the COVID-19 pandemic | International Journal of Africa Nursing Sciences | 10.1016/j.ijans.2022.100407 | Ethiopia | Observational cross-sectional study | Rural and urban hospitals in Ethiopia | August 2020 - September 2020 | Prospective | - Predictors of adherence to exercise recommendations | Interrogation of patient records / structured questionnaire used for data extraction | Adult type 2 diabetes patients attending the hospital diabetes follow-up clinic | 576 | - 26.4% only adhered to physical exercise recommendations. - Rural residence was associated with higher odds of adherence to physical exercise recommendations (AOR: 1.95, 95% CI: 1.16,3.27, *p<0.05*) - Being female was associated with higher odds of physical exercise adherence (AOR: 1.86, 95%CI, 1.27-2.72, *p<0.01*) |
| 13 | Bepouka BI, et al. | 2020 | Predictors of mortality in COVID-19 patients at Kinshasha University Hospital, DRC from March to June 2020 | Pan African Medical Journal | 10.11604%2Fpamj.2020.37.105.25279 | Democratic Republic of Congo | Observational cohort | Urban hospital in Kinshasa, Democratic Republic of Congo | March 2020 - June 2020 | Retrospective | - Characteristics of cases - Predictors of survival and mortality | Interrogation of medical records | Hospitalised patients with RT-PCR confirmed COVID-19 | 141 | - 17% of COVID-19 hospitalised patients had diabetes. - Patients with diabetes had reduced COVID-19 survival, *p=0.015* |
| 14 | Boulle, et al. | 2021 | Risk factors for Coronavirus Disease 2019 (COVID-19) death in a population cohort study from western Cape province, South Africa | Clinical Infectious Diseases | 10.1093%2Fcid%2Fciaa1198 | South Africa | Observational cohort | Rural and urban hospitals in West Cape Province, South Africa | March 2020 - June 2020 | Retrospective | - Predictors of COVID-19 death | Interrogation of patient records / structured questionnaire used for data extraction | Patients with PCR-diagnosed COVID-19 | 3,460,932 | Diabetes was associated with COVID-19 death with the risk of death increasing with higher HbA1c values: <7% (HR 1.44, 95% CI: 1.06-1.96, *p=0.02*), 7%<9% (HR 1.81, CI:1.39-2.35, *p<0.001*), ≥9% (HR 1.60, CI: 1.27-2.0, *p<0.001*) all vs those without diabetes. |
| 15 | Poaty H, et al. | 2021 | Diabetes and COVID-19 in Congolese patients | African Health Sciences | 10.4314%2Fahs.v21i3.18 | Congo | Observational cross-sectional study | Urban hospital in The Congo | March 2020 - August 2020 | Retrospective | - Characteristics of COVID-19 death | Photographing of clinical notes and remote clinical data entry / REDCap | Patients with pre-existing diabetes infected with SARS-CoV 2 | 30 | Diabetes patients with COVID-19 had a mortality rate of 36.7% |
| 16 | Ikram AS & Pillay S | 2022 | Hyperglycaemia, diabetes mellitus and COVID-19 in a tertiary hospital in KwaZulu Natal | Journal of Endocrinology, Metabolism and Diabetes of South Africa | 10.1080/16089677.2021.1997427 | South Africa | Observational cohort | Urban hospital in KwaZulu Natal, South Africa | June 2020 - September 2020 | Retrospective | - Predictors of mortality | Interrogation of secondary data from a medical insurance scheme | Hospitalised patients >13years with laboratory-confirmed SARS-CoV 2 infection | 236 | - 50% of those admitted with hyperglycaemia having no history of diabetes died - 26.6% of those living with diabetes (pre-existing or newly diagnosed) died. - patients presenting with admission hyperglycaemia had higher odds of death (OR:4.24, 95%CI:1.12-16) - Patients with diabetes had higher odds of dying compared to those with no diabetes (OR: 1.97, 95% CI:0.99-3.89) |
| 17 | Leulseged TW, et al. | 2022 | COVID-19 severity and associated factors among Ethiopian patients: a study of the millennium COVID-19 care centre | PLOS ONE | 10.1371/journal.pone.0262896 | Ethiopia | Observational cross-sectional study | Urban hospital in Ethiopia | June 2020 - August 2020 | Prospective | - Characteristics of cases - Predictors of COVID-19 severity | Face-to-face interview / interview guide and standardised investigation form | Patients admitted with RT-PCR confirmed diagnosis of COVID-19 | 686 | - 16.6% of cases had diabetes. - Diabetes had higher odds of COVID-19 severity than those who had no diabetes (AOR: 3.93, 95% CI: 1.96,7.85) - 29.9% of the severe cases had diabetes |
| 18 | Leulseged TW., et al. | 2021 | Characteristics and outcome profile of hospitalised African patients with COVID-19: the Ethiopian context | PLOS ONE | 10.4269%2Fajtmh.21-1270 | Ethiopia | Observational cohort | Rural and urban hospitals in Ethiopia | July 2020 - September 2020 | Prospective | - Characteristics of cases - Predictors of recovery | Review of medical records / Checklist | Patients admitted with RT-PCR confirmed diagnosis of COVID-19 | 1,345 | - 13.7% of cases had diabetes. - Diabetes had a higher median duration of recovery (15 days) than those with no diabetes. - Having diabetes was associated with 45.1% (p=0.005) lower odds of achieving clinical recovery compared to those without diabetes (AOR=0.549, 95% CI:0.337,0.894; p=<0.05). |
| 19 | Adjei P, et al. | 2020 | Clinical characteristics of COVID-19 patients admitted at the Korle-Bu Teaching hospital, Accra, Ghana | Ghana Medical Journal | 10.4314%2Fgmj.v54i4s.6 | Ghana | Observational cross-sectional study | Urban hospital in Accra, Ghana | April 2020 - June 2020 | Retrospective | - Clinical characteristics | Extraction of data from patient folders and patient survey / standardised data collection sheet and survey questionnaire | Hospital-admitted COVID-19 diagnosed patients | 50 | - 42% of cases had diabetes. - 90.5% of those with complications had diabetes. - 23.8% of those with diabetes died |
| 20 | van der Westhuizen JN, et al. | 2021 | Low mortality of people living with diabetes mellitus diagnosed with COVID-19 and managed at a field hospital in Western Cape Province, South Africa | South African Medical Journal | 10.7196/samj.2021.v111i10.15779 | South Africa | Observational cross-sectional study | Rural hospital in Western Cape Province, South Africa | June 2020 - August 2020 | Retrospective | - Characteristics of cases - Predictors of death | Extraction of patient data / structured extraction checklist | Patient admitted to the hospital with confirmed COVID-19 and pre-existing or newly diagnosed diabetes | 1,447 | - 5.3% of persons with diabetes died and 6.7% were referred for higher specialised care. - 86.5% had HbA1c >7%, median (IQR): 10% (8-12%). - Being male (OR=2.05, 95%CI=1.07,3.93) and on insulin (OR=2.25, 95% CI=1.05,4.85) was associated with higher odds of death |
| 21 | Delobelle PA, et al. | 2022 | Non-communicable disease care and management in two sites of the Cape town Metro during the first wave of COVID-19: a rapid appraisal | African Journal of Primary Health Care & Family Medicine | 10.4102/phcfm.v14i1.3215 | South Africa | Mixed-methods study | Urban primary settings in South Africa | October 2020 - November 2020 | Prospective | - Appraisal of care and management | Patients interviews and review for patient records | primary care facility managers, professional healthcare workers, community health workers, patients living with NCDs (type 2 diabetes and hypertension) | 28 | - Cancellation of routine non-communicable disease clinic services and chronic patient 'clubs' - Reduced availability of healthcare workforce - Introduction of clinic booking for clinics that improved clinic congestion. - Home delivery of medication using community health workers was adopted to decongest health facilities. - Improved performance of community health workforce - Patient stigma associated with patient home visits and deliveries. - General increase in workload among the health workforce. - General decrease in the number of NCD patients visiting the facility compared with prior to COVID-19 period. - Higher proportion of patients with uncontrolled diabetes. |
| 22 | Crankson S, et al. | 2022 | Determinants of COVID-19-related length of hospital stays and long COVID in Ghana: a cross-sectional analysis | International Journal of Environmental Research and Public Health | 10.3390/ijerph19010527 | Ghana | Observational cross-sectional study | Urban hospital in Ghana | March 2020 - August 2021 | Retrospective | - Characteristics of cases - Predictors of long COVID and hospitalisation | Patient medical records review | PCR confirmed COVID-19 patients | 2,334 | - Comorbid diabetes occurred in 2% of patients - Long COVID occurred in 4.3% of persons with diabetes - Diabetes was associated with longer LOS (B=1.37, 95% CI=0.99-1.88, p <0.05) |
| 23 | Ephraim RKD, et al. | 2021 | Psychological impact of COVID-19 on diabetes mellitus patients in Cape Coast, Ghana: a cross-sectional study | Pan African Medical Journal | 10.11604%2Fpamj.2021.40.76.26834 | Ghana | Observational cross-sectional study | Rural and urban hospitals in Cape Coast, Ghana | June 2020 - September 2020 | Prospective | - Characteristics of cases | Semi-structured qualitative interviews (guide) | Diabetes patients aged 20 years and over | 157 | - 82.8% of PWDM aged over 20 were ‘more careful about taking medication than usual’, - 33.8% worried “about people with diabetes being characterised as a risk group”. - 42% expressed worry “they would be overly affected if infected with coronavirus due to diabetes” - 49.7% worried about ‘not being able to manage diabetes if infected with coronavirus’ - 42% reported to have eaten less than usual |
| 24 | Habineza JC, et al. | 2021 | Perceived impact of the COVID-19 pandemic on young adults with type 1 diabetes in Rwanda | Pan African Medical Journal | 10.11604/pamj.2021.40.252.28899 | Rwanda | Observational cross-sectional study | Rural and urban communities in Rwanda | June 2020 - September 2020 | Prospective | - Pandemic experiences and challenges - Coping mechanisms | hospital patient records | Young adults with type 1 diabetes | 52 | - 80.8% reported a drop in family income - 57.7% reported a reduction in meal frequency - 43.1% reported reduced physical activity - Increased episodes of hypoglycaemia during the lockdown - Patients faced problems with law enforcement when trying to access their diabetes supplies and attend diabetes healthcare appointments, in ways they had not previously experienced - Reduced use of motorised transport and increased footing to diabetes care centres during the pandemic |
| 25 | Baguma S, et al. | 2022 | Factors associated with mortality among the COVID-19 patients treated at Gulu Regional Referral Hospital: a retrospective study | Frontiers in Public Health | 10.3389/fpubh.2022.841906 | Uganda | Observational cohort | Urban Hospital in Northern Uganda | March 2020 - October 2021 | Retrospective | - Characteristics of cases - Predictors of mortality | close-ended structure questionnaire | Hospitalised patients with confirmed COVID-19 | 664 | - 34.4% of COVID-19 deaths occurred in patients with diabetes. - Diabetes was associated with higher odds of death compared to those who had no diabetes (AOR=9.014, 95% CI=1.726 - 47.067) |
| 26 | Iroungou BA, et al. | 2021 | Demographic clinical characteristics associated with severity, clinical outcomes and mortality of COVID-19 infection in Gabon | JAMA Network Open | 10.1001/jamanetworkopen.2021.24190 | Gabon | Observational cross-sectional study | Urban hospital in Lebreville, Gabon | March 2020 - June 2020 | Retrospective | - Characteristics of severe COVID-19 | Self-administered questionnaire | COVID-19 hospitalised patients | 837 | 16.1% patients with a history of diabetes had severe COVID-19 |
| 27 | Awucha EN, et al. | 2020 | Impact of the COVID-19 pandemic on consumers' access to essential medicines in Nigeria | American Journal of Tropical Medicine and Hygiene | 10.4269%2Fajtmh.20-0838 | Nigeria | Observational cross-sectional study | Rural and urban community settings in Nigeria | May 2020 - June 2020 | Prospective | - Impact on essential medicine access | Data abstraction tool | Persons aged 15 years and older | 374 | - The proportion of patients with difficulty in accessing essential medicines during the COVID-19 pandemic was significantly higher than before the pandemic (29.6% vs 5.6%, p<0.001) |
| 28 | Kaswa RP. & Meel B. | 2021 | A Study on the Characteristic Features of COVID-19 Deaths in a Regional Hospital in Mthatha in the Eastern Cape, South Africa. | Indian Journal of Forensic Medicine & Toxicology | 10.4102%2Fsafp.v63i1.5253 | South Africa | Observational cross-sectional study | Urban hospital in Eastern Cape, South Africa | July 2020 - January 2021 | Retrospective | - Characteristics of COVID-19 deaths | Patient medical records | Records of patients who died of COVID-19 | 100 | 37% of patients had diabetes |
| 29 | Usui R, Kanamori S, Aomori M. & Watabe S. | 2022 | Analysis of COVID-19 mortality in patients with comorbidities in Côte d'Ivoire | Journal of Public Health in Africa | 10.4081%2Fjphia.2022.1748 | Côte d'Ivoire | Observational cross-sectional study | Rural and urban hospitals in Cote d’Ivoire | March 2020 - July 2020 | Retrospective | - Comorbidities associated with COVID-19 deaths | Self-administered web-based electronic questionnaire | COVID-19 infected persons | 67 | 45% of COVID-19 deaths occurred in patients with diabetes |
| 30 | Tagoe ET, Nonvignon J, van Der Meer R, Megiddo I, & Godman B. | 2023 | Challenges to the delivery of clinical diabetes services in Ghana created by the COVID-19 pandemic | Journal of Health Services Research & Policy | 10.1177/13558196221111708 | Ghana | Qualitative study | Rural and urban hospitals in Ghana | November 2020 - February 2021 | Prospective | - COVID-19 impact on diabetes service delivery | Excel spreadsheet used to manually collect data from clinical health records | Healthcare professionals and health facility administrators | 18 | Themes:   - high medicine and service costs and medicine shortages (disruption in supply chain, rationing, increased pricing of medicines) - poor patient information management (substandard anthropometric procedures, increase in records with missing data, misplacement of patient record files) - few trained healthcare providers (COVID-19 treatment prioritisation, patient rejection of referrals, high patient load) - low healthcare provider motivation (unsupportive management) - service organisation challenges (extended patient reviews, clinic overcrowding, increased clinic waiting times) - national health policy-related concerns (policy restrictions could not allow flexibility in planning and cost sharing) |
| 31 | Sikhosana ML, Jassat W & Makatini Z. | 2022 | Characteristics of hospitalised COVID-19 patients during the first two pandemic waves, Gauteng | Southern African Journal of Infectious Diseases | 10.4102/sajid.v37i1.434 | South Africa | Observational cross-sectional study | Rural and urban hospitals in Gauteng, South Africa | March 2020 - March 2021 | Retrospective | - characteristics of cases | Review of published health reports | SARS CoV 2 admitted patients | 1,861 | 21.6% of cases had diabetes |
| 32 | Elijah IM, et al. | 2022 | Characterization and determinant factors of critical illness and in-hospital mortality of COVID-19 patients: A retrospective cohort of 1,792 patients in Kenya | Biosafety and Health | 10.1016/j.bsheal.2022.06.002 | Kenya | Observational cohort | Rural and urban hospitals in Kenya | March 2020 - April 2021 | Retrospective | - Predictors of hospitalisation and survival - Prevalence of diabetes comorbidity | Qualitative semi-structured interviews / topic guide | Admitted COVID-19 patients | 1,792 | - 5.4% cases had diabetes - Diabetes was a significant predictor of ICU admissions (aOR: 3.30, 95%CI: 1.94 - 560, p<0.0001) - Diabetes was significantly associated with less survival probability compared to those without diabetes (p<0.0001) |
| 33 | Hardy YO, et al. | 2023 | Clinical and laboratory profile and outcomes of hospitalized COVID-19 patients with type 2 diabetes mellitus in Ghana - A single-center study. | Endocrinology, Diabetes & Metabolism | 10.1002/edm2.391 | Ghana | Observational cross-sectional study | Urban hospital in Ghana | March 2020 - October 2020 | Retrospective | - Prevalence of comorbid diabetes - Predictors of hospitalisation | Data records of the national COVID-19 DATCOV surveillance system | Adult patients hospitalised with COVID-19 | 175 | - 36.6% patients had type 2 diabetes - No significant difference in COVID-19 severity and duration of hospitalisation between patients with diabetes and those without |
| 34 | Huluka DK, et al. | 2022 | Clinical Characteristics and Treatment Outcomes of COVID-19 Patients at Eka Kotebe General Hospital, Addis Ababa, Ethiopia | American Journal of Tropical Medicine and Hygiene | 10.4269%2Fajtmh.21-1270 | Ethiopia | Observational cross-sectional study | Urban hospital in Addis Ababa, Ethiopia | March 2020 - September 2020 | Retrospective | - Clinical characteristics of cases | Interrogation of patient records / structured questionnaire used for data extraction | SARS CoV 2 positive patients aged ≥18 | 463 | - 20.7% of cases had diabetes. - 33.1 % of those who experienced severe COVID-19 had diabetes. - 35.8% of COVID-19 deaths had diabetes |
| 35 | Nyasulu PS, et al. | 2022 | Clinical characteristics associated with mortality of COVID-19 patients admitted to an intensive care unit of a tertiary hospital in South Africa | PLOS ONE | 10.1371/journal.pone.0279565 | South Africa | Observational cross-sectional study | Urban hospital in South Africa | March 2020 - November 2020 | Prospective | - Characteristics of cases and outcomes | Interrogation of medical records | ICU admitted COVID-19 patients aged ≥18 | 413 | - 51% of patients had comorbid diabetes. - 66% of patients with comorbid diabetes died |
| 36 | Solanki G, et al. | 2022 | COVID-19 hospitalization and mortality and hospitalization-related utilization and expenditure: analysis of a South African private health insured population | PLOS ONE | 10.1371%2Fjournal.pone.0268025 | South Africa | Observational cross-sectional study | Rural and urban communities in South Africa | March 2020 - June 2021 | Retrospective | - Risk of hospitalisation | Interrogation of patient records / structured questionnaire used for data extraction | Private health insurance patients who tested positive for COVID-19 | 188,292 | Diabetes was associated with high risk for hospitalisation (OR 3.6; 95% CI 3.27 - 3.94) |
| 37 | Diarra M, et al. | 2022 | First wave COVID-19 pandemic in Senegal: Epidemiological and clinical characteristics | PLOS ONE | 10.1371/journal.pone.0274783 | Senegal | Observational cross-sectional study | Rural and urban hospitals in Senegal | March 2020 - October 2020 | Prospective | - Clinical characteristics - Predictors of mortality | Photographing of clinical notes and remote clinical data entry / REDCap | Community population | 67,608 | - 38.2% of cases had diabetes - Relative risk for COVID-19 mortality was high in persons with comorbid diabetes (aRR=1.31, 95%CI=0.77-2.23, *p<0.001*) |
| 38 | Tolossa T, et al. | 2022 | Incidence and predictors of diabetes mellitus among severe COVID-19 patients in western Ethiopia: a retrospective cohort study | Journal of Endocrinology, Metabolism and Diabetes of South Africa | 10.1080/16089677.2022.2144016 | Ethiopia | Observational cohort | Urban hospital western Ethiopia | September 2020 - June 2021 | Retrospective | - Clinical characteristics - Predictors of onset diabetes | Interrogation of secondary data from a medical insurance scheme | Severe COVID-19 hospital-admitted patients | 304 | - Incidence of diabetes among patients was 14.5%. - Overall diabetes incidence rate at the end of follow-up (34 days) was 13.7/1,000 person day's observation (95% CI 10.2, 18.4) - Median occurrence of diabetes was 11 days (95% CI: 7, 13) - Risk of developing diabetes increased for the first 20 days and was constant thereafter (Kaplan-Meier survival estimate) - Predictors of Diabetes included older age >41 years (AHR = 2.54, 95% CI: 1.15, 5.57, compared to <25years, p=0.02), residing in urban settings (AHR = 2.49, 95% CI: 1.12, 5.52, compared to rural, p=0.02), being admitted within 48 hours of clinical manifestation compared to >48hours (0.49, 95% CI: 0.23,0.96 ref.≤48hrs, p=0.04) |
| 39 | David NJ, et al. | 2022 | Measuring the impact of community-based interventions on type 2 diabetes control during the COVID-19 pandemic in Cape Town - A mixed methods study. | South African Family Practice | 10.4102/safp.v64i1.5558 | South Africa | Mixed-methods study | Rural community in South Africa | September 2020 - December 2020 | Prospective | - Home delivery of medicines | Face-to-face interview / interview guide and standardised investigation form | Type 2 diabetes patient attending routine care | 544 | - overall, HDM resulted in 0.46% reduction in HbA1c compared to non-HDM (p<0.01) - Patients perceived HDM as timesaving. - Patients perceived HDM as reducing exposure to coronavirus infection |
| 40 | Sane AH, et al. | 2022 | New Onset of Diabetes Mellitus and Associated Factors among COVID-19 Patients in COVID-19 Care Centers, Addis Ababa, Ethiopia 2022 | Journal of diabetes research | 10.1155/2022/9652940 | Ethiopia | Observational cross-sectional study | Urban hospitals in Addis Ababa, Ethiopia | September 2020 - September 2021 | Retrospective | - Prevalence of new-onset diabetes - Predictors of new-onset diabetes | Review of medical records / Checklist | COVID-19 admitted patients with diagnosed diabetes | 244 | - 31.1% of COVID-19 patients had onset Diabetes (95% CI: 25.4, 37.4) - Males were more likely to develop new onset diabetes than females (aOR=2.9, 95%CI:1.2,7.1, p=0.018) |
| 41 | Jassat W, et al. | 2022 | Undiagnosed comorbidities among individuals hospitalised with COVID-19 in South African public hospitals | South African Medical Journal | 10.7196/samj.2022.v112i9.16417 | South Africa | Observational cross-sectional study | Rural and urban hospital in South Africa | November 2020 - June 2021 | Prospective | - Prevalence of diabetes | Extraction of data from patient folders and patient survey / standardised data collection sheet and survey questionnaire | COVID-19 hospitalised patients | 3,217 | - 26.6% had self-reported diabetes - 7.3% of diabetes case were new diagnoses |
| 42 | Mengist B, Animut Z & Tolossa T. | 2022 | Incidence and predictors of mortality among COVID-19 patients admitted to treatment centers in North West Ethiopia; A retrospective cohort study, 2021 | International Journal of Africa Nursing Sciences | 10.1016/j.ijans.2022.100419 | Ethiopia | Observational cohort | Rural and urban hospitals in Northwest Ethiopia | March 2020 - March 2021 | Retrospective | - Prevalence of diabetes - Predictors of mortality | Extraction of patient data / structured extraction checklist | COVID-19 hospitalised patients | 552 | - 4% of patients had comorbid diabetes. - patients with diabetes had 8 times higher hazard mortality than those without diabetes (AHR: 8.1, 95% Ci:2.9, 22.4, *p<0.001*) |
